# Inhibition of NF-κB pathways alleviates hydrocephalus via modulation of choroid plexus epithelium inflammation in a rat intraventricular hemorrhage model

**DOI:** 10.1371/journal.pone.0336346

**Published:** 2025-11-21

**Authors:** Tong Sun, Yikai Yuan, Yicheng Zhou, Ke Wu, Chao You, Junwen Guan

**Affiliations:** 1 Department of Neurosurgery, West China Hospital, Sichuan University, Chengdu, Sichuan, P.R China; 2 Department of Neurosurgery, Xichang People’s Hospital, Liangshan, Sichuan, P.R China; Saitama Medical Center: Saitama Ika Daigaku Sogo Iryo Center, JAPAN

## Abstract

**Backgrounds:**

Post-hemorrhagic hydrocephalus (PHH) is a serious complication following hemorrhagic events due to cerebrospinal fluid (CSF) pathway disorders. We explore the Nuclear Factor κB (NF-κB) signaling pathway’s involvement in choroid plexus epitheliums (CPEs) inflammation and hydrocephalus, aiming to identify new therapeutic targets for managing PHH.

**Materials and Methods:**

Adult male Sprague-Dawley rats were used to establish an intraventricular hemorrhage (IVH) autologous-blood model. Rats were randomly assigned to four groups: artificial cerebrospinal fluid (aCSF), IVH, IVH + TNF-α inhibitor, and IVH + NF-κB inhibitor. CSF secretion rates, lateral ventricular volumes, and inflammatory cytokine levels in CSF were measured at 3, 7, and 14 days post-modeling. Western blotting and immunofluorescence were used to analyze NF-κB pathway activation and the related inflammatory markers, including NF-κB, TNF-α, Illinois, Na+-K+-Cl− co-transporter 1 (NKCC1), aquaporin-1 (AQP1), and aquaporin-4 (AQP4),

**Results:**

TNF-α and NF-κB inhibitors effectively reduce lateral ventricular enlargement and CSF secretion rates following IVH in rats. The concentration of TNF-α in the IVH group was significantly higher than in the aCSF group as well as the two inhibitor groups. On days 3 and 7 post-modeling, Western blot and immunofluorescence analyses revealed altered expression of pNF-κB (p65), and proteins in CPEs across groups, with TNF-α and NF-κB inhibition reducing pNF-κB and levels. Illinois and NKCC1 changes were tissue,

**Conclusions:**

NF-κB activation post-IVH drives CPEs inflammation, increases CSF production, and contributes to hydrocephalus formation. Targeting the NF-κB pathway offers a promising strategy for the treatment of PHH.

## Introduction

In early research, based on the classical cerebrospinal fluid (CSF) circulation theory, it was widely accepted that post-hemorrhagic hydrocephalus (PHH) was primarily caused by an obstruction of CSF pathways within the ventricles and/or impaired CSF absorption due to dysfunction of the arachnoid granulations. Multiple studies have supported this hypothesis. One such study found that post-hemorrhagic thickening of the leptomeninges led to the obstruction of the fourth ventricular outlet, resulting in hydrocephalus, referred to as “fourth ventricular” PHH [1]. Other studies have reported that following intraventricular hemorrhage (IVH), blood and its breakdown products can obstruct CSF pathways, such as the cerebral aqueduct, thereby impeding CSF circulation and leading to PHH [2]. Furthermore, subacute or chronic communicating hydrocephalus often follows hemorrhagic events. It has been proposed that the development of communicating hydrocephalus after IVH is primarily related to arachnoid granulation dysfunction. Microthrombi and blood clots generated from the hemorrhage may obstruct the villi of the arachnoid granulations, impairing CSF reabsorption. Another perspective suggests that post-hemorrhagic inflammation in the periventricular ependyma may cause structural damage, potentially hindering normal CSF flow, and leading to abnormal CSF accumulation within the ventricular system [3,4].

However, these theories neglect the role of increased CSF production in the development and progression of PHH, and they overlook critical foundational and clinical evidence concerning the involvement of the choroid plexus epitheliums (CPEs) inflammatory response in PHH [5]. Additionally, these theories do not account for the functional differences in arachnoid granulations across age groups. While arachnoid granulations are believed to absorb the majority of CSF in adults, they are either absent or underdeveloped in human infants and most animal models of PHH [6]. Moreover, other structures, including the ventricular ependyma, perineural spaces, leptomeninges, lymphatic vessels, and nasal mucosa, also contribute to CSF absorption beyond the arachnoid granulations [7–9]. Therefore, current evidence suggests that arachnoid granulation dysfunction is not the dominant factor in the pathophysiology of PHH. Recent studies have shown that injecting blood metabolites into the lateral ventricles of animals induces CPEs inflammation and hydrocephalus. These findings indicate that post-hemorrhagic CPEs inflammation may play a significant role in PHH, and mitigating this inflammation may serve as an effective therapeutic strategy [10,11].

The role of inflammation in the pathogenesis of PHH has been studied as early as 1840, and clinical data have since validated this concept [7,12]. For example, in both infants and adults with hemorrhagic events, elevated levels of inflammatory markers such as interleukin-6 (IL-6), IL-4, tumor necrosis factor-α (TNF-α), transforming growth factor-β1 (TGFβ1), and others in CSF and peripheral blood have been correlated with the incidence and severity of secondary hydrocephalus [13–16]. Neuropathological examinations of brain tissue from infants with PHH have also revealed signs of neuroinflammation, including microglial activation and reactive gliosis [17]. A randomized, double-blind, placebo-controlled clinical trial demonstrated that dexamethasone treatment significantly reduced the incidence of hydrocephalus in adult patients with tuberculous meningitis [18]. Additionally, another clinical study suggested that increasing the dexamethasone dosage following aneurysmal subarachnoid hemorrhage might reduce the risk of hydrocephalus [19,20]. Experimental research has also confirmed the pivotal role of inflammation in the development of PHH. In rodent PHH models, inflammatory responses were observed in the choroid plexus and ependyma of the lateral ventricles following hemorrhage. Histological examinations of brain tissue revealed leukocyte infiltration, microglial activation, and reactive gliosis [21]. In other animal models, dexamethasone administration was found to inhibit CSF production at the CPEs [22]. Consequently, subsequent research has focused on exploring the pathophysiological mechanisms underlying CPEs inflammation after hemorrhage. Simard et al. [11] employed an autologous blood injection model in rats and found significant ventricular enlargement two days post-IVH, along with pronounced activation of the nuclear factor κB (NF-κB) signaling pathway in the choroid plexus and ependyma. This activation triggered an inflammatory response that contributed to the development of hydrocephalus.

Nevertheless, the mechanisms underlying NF-κB pathway activation following hemorrhagic events and its role in mediating the development of hydrocephalus remain unclear. This study aims to investigate the role of CPEs inflammation-regulating pathways in the pathogenesis of hydrocephalus and elucidate the underlying pathophysiological mechanisms using a PHH rat model.

## Materials and methods

### Experimental animals and modeling

All the animal studies in this work were approved by the Animal Care and Use Committee of Sichuan University (Chengdu, Sichuan, China). This study utilized clean-grade adult male Sprague-Dawley (SD) rats, weighing between 200 and 250 g, purchased from Chengdu Dashuo Laboratory Animal Co., Ltd. The rats were housed in an artificial environment with a controlled light-dark cycle, free access to food and water, with a 12-hour light-dark cycle, a room temperature of 23°C, and appropriate humidity levels. Totally 106 rats were applied in our research.

**Fig 1:**
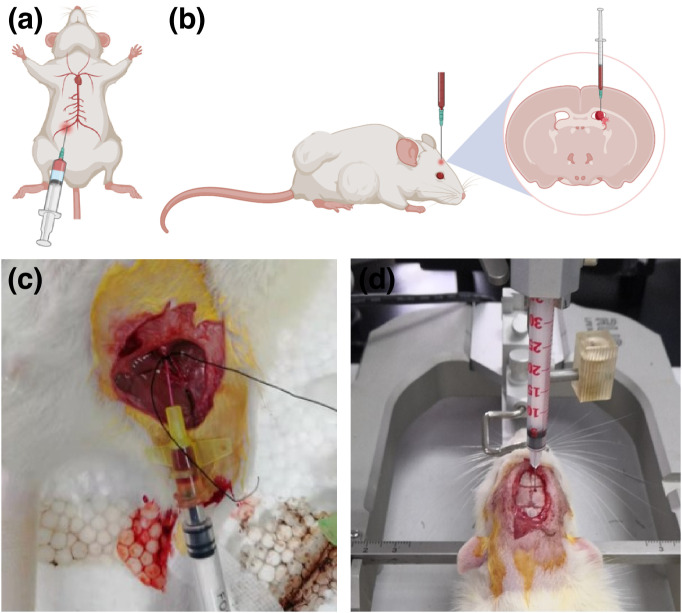
Model Establishment. (a) Schematic; (b) experimental illustration. The skin on the medial side of the rat’s left thigh was exposed and secured. After incising the skin and carefully dissecting layer by layer, the left femoral artery was isolated. A 500 μL syringe was used to puncture the artery and withdraw 200 μL of blood (without any anticoagulant). Using the bregma as the reference point (0.6 mm posterior and 1.6 mm to the right), 200 μL of freshly collected blood was uniformly and slowly injected into the right lateral ventricle at a rate of approximately 40 μL/min. After completing the injection, the needle tip was maintained in place for 10 minutes before being gradually withdrawn. (a) Schematic; (b) Experimental Illustration.

The IVH model was established by injecting autologous femoral arterial blood into the lateral ventricle, as shown in Fig 1. The baseline body weight of each rat was recorded prior to the procedure. Anesthesia was induced via intraperitoneal injection of 10% chloral hydrate (3 μl of chloral hydrate per gram of body weight). After successful anesthesia, the rats’ heads and inner thighs were shaved, and they were secured in a stereotaxic apparatus. Both the head and inner thigh were disinfected three times with a 2% povidone-iodine solution (Ailek).

A midline scalp incision, approximately 1 cm long, was made 1 mm behind the eye to expose the bregma. Any remaining periosteum was removed with cotton swabs soaked in 3% hydrogen peroxide. Using the bregma as the reference point, a position 0.6 mm posterior and 1.6 mm to the right was marked on the skull as the location of the right lateral ventricle. A handheld cranial drill was used to create a burr hole approximately 1 mm in diameter, taking care not to damage the dura mater or brain tissue.

The left inner thigh skin was exposed, incised, and dissected layer by layer to isolate the left femoral artery. A 500 μl syringe was used to withdraw 200 μl of blood (without anticoagulant). The syringe was immediately mounted on the stereotaxic apparatus, and the needle was inserted vertically into the burr hole to a depth of 4.5 mm. The freshly collected blood was injected into the right lateral ventricle at a controlled rate of approximately 40 μl per minute. After completing the injection, the needle was left in place for 10 minutes before being slowly withdrawn. The burr hole was sealed with bone wax, and the surgical incision was sutured. The head and inner thigh were disinfected with 2% povidone-iodine and 75% ethanol. The rats were then placed on a heating pad with free access to food and water.

At the end of our experiments, the rats were anaesthetized and humanly sacrificed by carbon dioxide.

### Experimental grouping

After one week of acclimatization, the rats were randomly divided into four groups: artificial CSF group (aCSF), IVH group, IVH + TNF-α inhibitor group, and IVH + NF-κB inhibitor group. In the IVH group, modeling was performed as described above. The aCSF group received 200 μl of sterile aCSF (Tocris) injected into the lateral ventricle using the same method. In the IVH + NF-κB inhibitor group, after IVH modeling, rats were immediately administered an intraperitoneal injection of 0.5 mL NF-κB inhibitor, pyrrolidinedithiocarbamate (PDTC, 100 mg/kg dissolved in saline). The injection was repeated every 8 hours with 0.25 mL administered each time, continuing for 48 hours post-hemorrhage. In the IVH + TNF-α inhibitor group, after IVH modeling, rats were immediately administered an intraperitoneal injection of 0.5 mL TNF-α inhibitor, R-7050 (EMD Biosciences, 15 mg/kg dissolved in saline), followed by additional injections every 12 hours, with 0.25 mL administered each time, continuing for 48 hours post-hemorrhage.

### Measurement of CSF secretion rate

**Fig 2:**
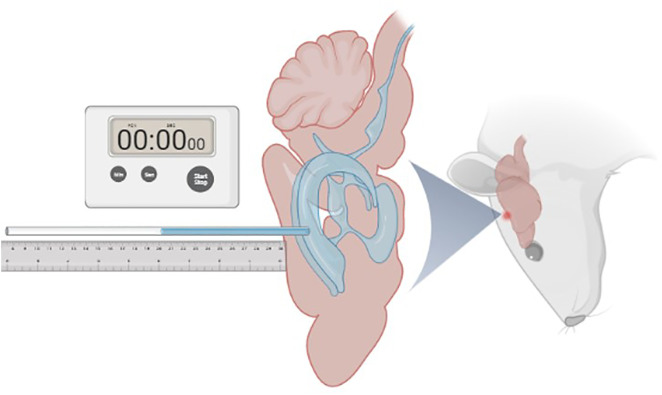
Measurement of CSF secretion rate in rats. To measure CSF secretion rate, the rat was positioned nose-down, perpendicular to the ground, with the mouth and nose securely fixed to maintain a vertical posture. Pre-warmed sterile mineral oil (100 μL, Sigma-Aldrich) at 37°C was gently injected into the fourth ventricle via the Magendie foramen to block the Sylvian aqueduct. After maintaining this position for 10 minutes, the needle tip was carefully withdrawn. A pre-marked capillary tube (inner diameter 1 mm) was positioned perpendicular to the cranial window and parallel to the ground. The tube was gently inserted 4 mm into the anterior horn of the lateral ventricle through the bone opening, allowing CSF to flow out (indicated by arrows). The CSF secretion rate was calculated by recording the volume of CSF collected (measured as the fluid volume in the capillary tube) and the time required for its outflow. CSF: cerebrospinal fluid.

The CSF secretion rate was measured on days 3, 7, and 14 post-modeling, following the methodology described in previous literature (Karimy et al., 2015), as illustrated in Fig 2. Rats were anesthetized with 10% chloral hydrate (30 μl/10 g) administered via intraperitoneal injection, and the surgical area was prepared. The rats were then secured in a stereotaxic apparatus. The head and inner thigh were disinfected with 2% povidone-iodine solution and 75% ethanol. A midline incision was made along the skull to expose the bregma and coronal suture. Cotton swabs soaked in 3% hydrogen peroxide were used to remove any remaining periosteum. Based on the rat brain atlas, the coordinates were determined as 0.8 mm posterior and 1.7 mm lateral to the bregma, marking the surface of the skull for the left lateral ventricle. A handheld cranial drill was used to create a burr hole approximately 1 mm in diameter, avoiding damage to the dura mater and brain tissue.

The rat’s hind limbs were elevated by 2 cm, and the incisor bar was removed. The rat’s head was rotated 90° on the ear bars, with the nose pointed downward, perpendicular to the ground, and the mouth and nose securely fixed in this vertical position. An incision was made at the occipital-cervical junction, and neck muscles were separated using retractors, followed by partial removal of ligaments. A fine insulin syringe was used to puncture the dura mater just below the midline, and 100 μL of sterile mineral oil preheated to 37°C (Sigma-Aldrich) was gently injected 5 mm into the fourth ventricle through the Magendie foramen to block the Sylvian aqueduct. After 10 minutes, the needle was gently withdrawn.

A capillary tube with pre-marked gradations (inner diameter 1 mm) was inserted vertically into the burr hole and gently advanced 4 mm into the anterior horn of the lateral ventricle, allowing CSF to flow out (the capillary tube must remain stable). The CSF secretion rate was calculated by measuring the volume of CSF that flowed out (based on the volume of fluid in the capillary tube) and the time required for its collection.

### Measurement of lateral ventricular volume

On days 3 and 7 post-modeling, the lateral ventricular volume was calculated using magnetic resonance imaging (MRI). Imaging was performed with a Siemens TRIO 3.0 T MRI scanner equipped with a specialized rat animal coil. Rats scheduled for MRI evaluation underwent pre-modeling and pre-grouping MRI scans to ensure a consistent baseline. During MRI scans, the rats were anesthetized with a 2% isoflurane/air mixture (flow rate: 30 mL/min). Once anesthetized, the rats were positioned prone and secured for imaging. The imaging protocol included T1-weighted imaging (TSE, TR = 400 ms, TE = 12.6 ms) and T2-weighted imaging (SE, TR = 4000 ms, TE = 96.7 ms). The field of view was 30 × 30 mm, with a matrix of 320 × 256 mm. A total of 24 coronal images (slice thickness: 1 mm) were obtained, covering the entire axis of the lateral ventricle.

All image analyses were conducted using Image J software. As previously described, the lateral ventricular volume was calculated from the T2-weighted images. The bilateral ventricles were outlined in 10 slices, and the area was measured. The ventricular areas from all slices were summed, representing the lateral ventricles, and multiplied by the slice thickness (1 mm) to obtain the lateral ventricular volume. The of ventricular volume was performed by blinded testers.

### Collection of CSF and measurement of inflammatory cytokines

**Fig 3:**
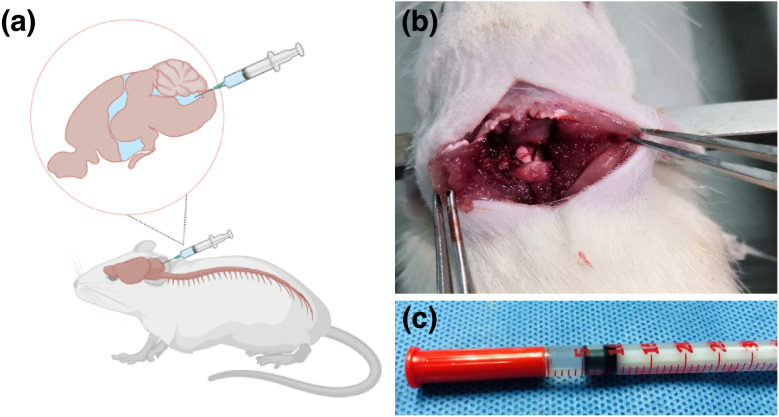
Collection of CSF. (a) Schematic illustration; (b) experimental illustration. An incision was made on the skin at the occipito-cervical junction. Using a retractor, the neck muscles were separated to expose the atlanto-occipital membrane located between the occipital ridge and the atlas vertebra. A 23-gauge insulin syringe (outer diameter 0.6 mm) was inserted at a 30° angle relative to the atlanto-occipital membrane and advanced approximately 1 mm. A total of 20 μL of CSF was aspirated and immediately transferred to an Eppendorf tube, which was stored at −80°C for further analysis. CSF: cerebrospinal fluid.

Following the methodology described in previous literature, CSF was collected from the cisterna magna (cerebellomedullary cistern), as illustrated in Fig 3 (Höistad et al., 2005). Rats were anesthetized with 10% chloral hydrate (30 μl/10 g) administered via intraperitoneal injection, and the surgical area was prepared. The rats were then secured in a stereotaxic apparatus with the head tilted downward. The skin was disinfected using 2% povidone-iodine solution and 75% ethanol. An incision was made at the occipital-cervical junction, and neck muscles were separated with retractors to expose the atlanto-occipital membrane between the occipital ridge and the atlas. A 23-gauge insulin syringe (outer diameter 0.6 mm) was inserted at a 30° angle through the atlanto-occipital membrane, penetrating approximately 1 mm. A total of 20 μl of CSF was aspirated and immediately transferred to an EP tube, which was stored at −80°C.

The concentration of inflammatory cytokines was measured using CSF collected from rats 3 days post-modeling. ELISA kits (Sigma-Aldrich, USA) were used to determine the concentrations of TNF-α (Catalog No. RAB0479), IL-1α (Catalog No. RAB0272), IL-1β (Catalog No. RAB0277), IL-6 (Catalog No. RAB0311), and IL-10 (Catalog No. RAB0246) in the CSF.

### Western blot Analysis

3 days and 7 days post-modeling, rats were sacrificed, and the choroid plexus and periventricular tissues were dissected under a microscope. The tissues were promptly transferred to a −80°C freezer for storage. Western blot (WB) was performed to detect NF-κB, IL-1β, Na+-K+-Cl− co-transporter 1 (NKCC1), aquaporin-1 (AQP1), and aquaporin-4 (AQP4). Since NF-κB becomes active only when phosphorylated in the nucleus, we measured phosphorylated NF-κB p65 (pNF-κB). The detailed experimental procedure is as follows:

### Immunofluorescence Detection

Choroid plexus were collected for the immunofluorescence detection. The tissue samples were wrapped in gauze and placed into numbered embedding cassettes. The following sequence was used for dehydration, clearing, and paraffin infiltration: 75% ethanol overnight; 95% ethanol I for 1.5 hours; 95% ethanol II for 1.5 hours; 100% ethanol I for 1.5 hours; 100% ethanol II for 1.5 hours; xylene I for 1 hour; xylene II for 1 hour; paraffin I at 58–60°C for 2 hours; paraffin II at 58–60°C for 2 hours. Embedding, sectioning, slide baking, deparaffinization, antigen retrieval, and result reading were performed in sequence. The sections were dried in an oven, and anti-fluorescence quenching mounting medium was applied for mounting. Images were captured at 400x magnification and analyzed using Image J software to calculate the percentage of the region of interest (ROI).

### Statistical analysis

All data were analyzed using SPSS version 19 (IBM, Armonk, NY). Quantitative data were presented as mean ± standard deviation. Considering the small sample size in each group and the non-normal distribution of the data, non-parametric tests were used for statistical analysis. The Kruskal-Wallis rank-sum test was performed, followed by pairwise comparisons to evaluate differences between groups. A p-value of less than 0.05 (two-tailed) was considered statistically significant.

## Results

### Changes in lateral ventricular volume

**Fig 4:**
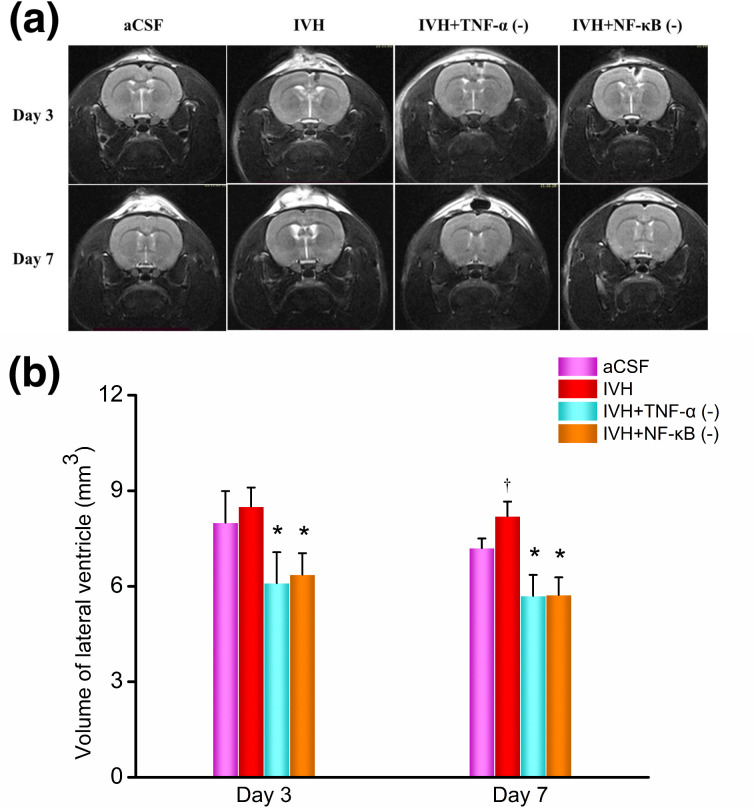
T2-weighted MRI imaging and lateral ventricle volume on days 3 and 7 post-modeling. n = 3; P < 0.05 vs. the IVH group; † P < 0.05 vs. the aCSF group. aCSF: artificial cerebrospinal fluid; IVH: intraventricular hemorrhage.

Lateral ventricular volumes were calculated from coronal T2-weighted MRI images of the rat brain. The coronal T2-weighted images and the calculated lateral ventricular volumes are shown in Fig 4. On day 3 post-modeling, the lateral ventricular volume in the aCSF group was 8.0 ± 1.0 mm³, and in the IVH group, it was 8.5 ± 0.6 mm³, with no statistically significant difference between the two groups (P = 0.451). The lateral ventricular volume in the IVH + TNF-α inhibitor group was 6.1 ± 1.0 mm³, significantly smaller than that of the IVH group (P = 0.013). Similarly, in the IVH + NF-κB inhibitor group, the lateral ventricular volume was 6.4 ± 0.7 mm³, which was also significantly smaller than that of the IVH group (P = 0.008).

On day 7 post-modeling, the lateral ventricular volume in the aCSF group was 7.2 ± 0.3 mm³, whereas it increased significantly in the IVH group to 8.2 ± 0.5 mm³ (P = 0.043). In contrast, the lateral ventricular volumes in the IVH + TNF-α inhibitor group and the IVH + NF-κB inhibitor group were both 5.7 ± 0.7 mm³ and 5.7 ± 0.6 mm³, respectively, significantly lower than those in the IVH group (P < 0.001 for both).

### CSF secretion rate

**Fig 5:**
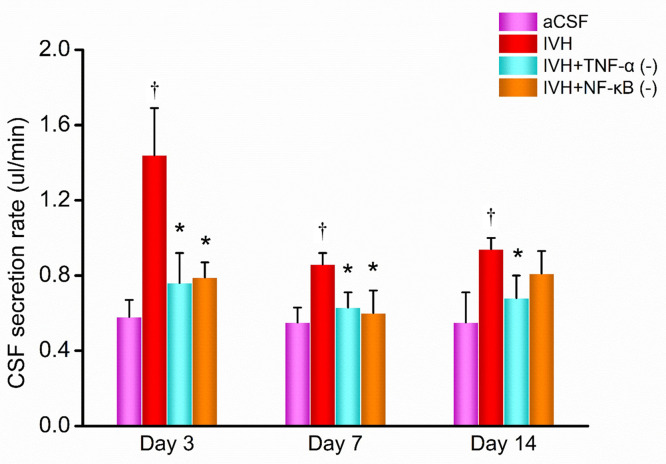
CSF secretion rate on days 3, 7, and 14 post-modeling. n = 3; P < 0.05 vs. the IVH group; e P < 0.05 vs. the aCSF group. aCSF: artificial cerebrospinal fluid; IVH: intraventricular hemorrhage.

After occluding the fourth ventricle outflow tract, the lateral ventricle was punctured using a capillary tube. The volume of CSF flowing out per unit time was used to represent the CSF secretion rate. The results are shown in Fig 5.

On day 3 post-modeling, the CSF secretion rate in the aCSF group was 0.58 ± 0.09 μL/min, while in the IVH group, it was significantly higher at 1.44 ± 0.25 μL/min (P < 0.001). The CSF secretion rate in the IVH + TNF-α inhibitor group was 0.76 ± 0.16 μL/min, which was lower than that of the IVH group (P = 0.001). In the IVH + NF-κB inhibitor group, the CSF secretion rate was 0.79 ± 0.08 μL/min, also significantly lower than the IVH group (P = 0.001).

On day 7 post-modeling, the CSF secretion rate in the aCSF group was 0.55 ± 0.08 μL/min, while in the IVH group, it increased significantly to 0.86 ± 0.08 μL/min (P = 0.003). In the IVH + TNF-α inhibitor group, the CSF secretion rate decreased to 0.63 ± 0.08 μL/min, significantly lower than the IVH group (P = 0.013). Similarly, the IVH + NF-κB inhibitor group exhibited a reduced CSF secretion rate of 0.60 ± 0.12 μL/min (P = 0.008), indicating the inhibitors’ potential to mitigate CSF hypersecretion.

On day 14 post-modeling, the CSF secretion rate in the aCSF group remained stable at 0.55 ± 0.16 μL/min. In contrast, the IVH group showed a significantly higher CSF secretion rate of 0.94 ± 0.08 μL/min (P = 0.004). The CSF secretion rate in the IVH + TNF-α inhibitor group was reduced to 0.68 ± 0.12 μL/min, significantly lower than that in the IVH group (P = 0.030). However, in the IVH + NF-κB inhibitor group, the CSF secretion rate was 0.81 ± 0.12 μL/min, which was not significantly different from the IVH group (P = 0.226).

### Levels of CSF cytokines

**Fig 6:**
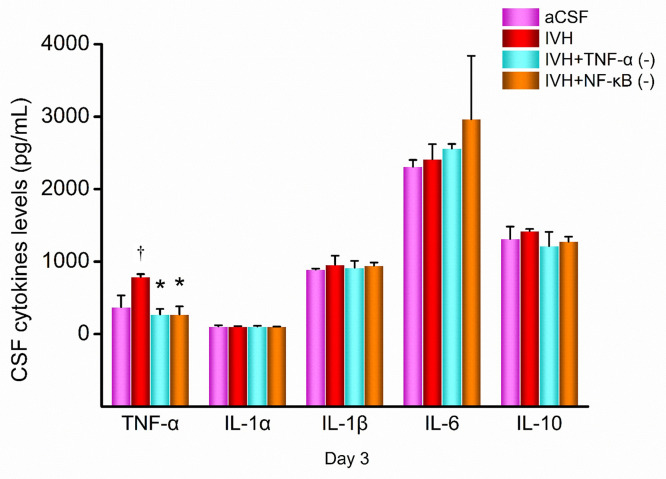
Concentration of inflammatory cytokines in CSF on day 3 post-modeling. aCSF group, n = 18; IVH group, n = 30; TNF-α inhibitor (-) group, n = 17; NF-κB inhibitor (-) group, n = 17. * P < 0.05 vs. the IVH group; e P < 0.05 vs. the aCSF group. CSF: cerebrospinal fluid; aCSF: artificial cerebrospinal fluid; IVH: intraventricular hemorrhage.

The levels of inflammatory cytokines in the CSF of rats were measured 3 days after hemorrhage using ELISA kits. The results are shown in Fig 6. **Briefly,** the concentration of TNF-α in the IVH group was significantly higher than that in the aCSF group. TNF-α inhibitor and NF-κB inhibitor significantly decreased the TNF-α peoduction after IVH treatment. However, no significant differences of IL-1α, IL-1β, IL-6, and IL-10 were observed by IVH treatment, TNF-α inhibitor or NF-κB inhibitor.

**TNF-α**: The concentration in the IVH group was 789 ± 39 pg/mL, which was significantly higher than in the aCSF group (371 ± 163 pg/mL) with a statistically significant difference (P = 0.001). In the IVH + TNF-α inhibitor group, the TNF-α concentration was 272 ± 75 pg/mL, significantly lower than in the IVH group (P < 0.001). The IVH + NF-κB inhibitor group had a TNF-α concentration of 513 ± 57 pg/mL, which was lower than the IVH group (P < 0.001) but higher than the IVH + TNF-α inhibitor group (P < 0.001).

**IL-1α**: The concentration in the IVH group was 107 ± 2 pg/mL, which did not differ significantly from the aCSF group (106 ± 16 pg/mL, P = 0.877). In the IVH + TNF-α inhibitor group, the IL-1α concentration was 106 ± 8 pg/mL, similar to the IVH group (P = 0.923). The IL-1α concentration in the IVH + NF-κB inhibitor group was 104 ± 1 pg/mL, showing no significant difference from the IVH group (P = 0.681).

**IL-1β**: The concentration in the IVH group was 957 ± 123 pg/mL, which was not significantly different from the aCSF group (889 ± 15 pg/mL, P = 0.335). In the IVH + TNF-α inhibitor group, the IL-1β concentration was 917 ± 93 pg/mL, similar to the IVH group (P = 0.566). The IL-1β concentration in the IVH + NF-κB inhibitor group was 944 ± 44 pg/mL, showing no significant difference from the IVH group (P = 0.850).

**IL-6**: The concentration in the IVH group was 2415 ± 206 pg/mL, which was not significantly different from the aCSF group (2309 ± 95 pg/mL, P = 0.782). In the IVH + TNF-α inhibitor group, the IL-6 concentration was 2561 ± 63 pg/mL, similar to the IVH group (P = 0.703). The IL-6 concentration in the IVH + NF-κB inhibitor group was 2965 ± 874 pg/mL, showing no significant difference from the IVH group (P = 0.175).

### Western blot analysis

The detailed West-blot results were shown in S1 Data and S1 File.

#### CPEs.

**Fig 7:**
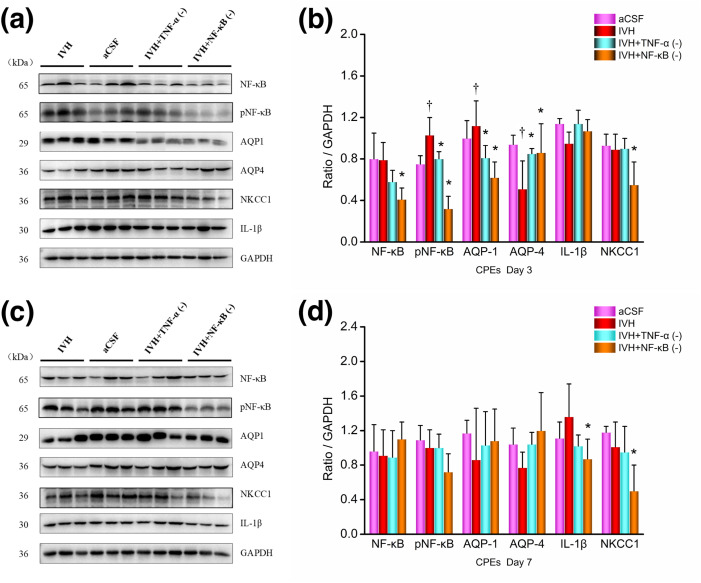
Western blot images and results of CPEs proteins. (a) + (b) Day 3; (c) + (d) Day 7. n = 3; *P < 0.05 vs. the IVH group; † P < 0.05 vs. the aCSF group. CPEs, choroid plexus epitheliums; CSF: cerebrospinal fluid; aCSF: artificial cerebrospinal fluid; IVH: intraventricular hemorrhage; TNF-α: tumor necrosis factor-α; pNF-κB: phosphorylated nuclear factor κB; AQP: aquaporins; NKCC1: Na ⁺ –K ⁺ –Cl ⁻ co-transporter 1.

The protein expression levels of various markers in CPEs 3 days and 7 days post-modeling were analyzed by immunoblotting, as shown in Fig 7. Protein expression levels were defined as the ratio of target protein grayscale values to the internal control protein GAPDH grayscale values.

On day 3 post-modeling, compared to the aCSF group, the IVH group showed increased expression of pNF-κB (p65, P = 0.019) and AQP1 (P = 0.036) proteins in the CPEs, while the expression of AQP-4 protein was decreased (P = 0.011). There were no statistically significant differences in the expression of NF-κB (P = 0.909), IL-1β (P = 0.218), or NKCC1 (P = 0.757) between the two groups. Compared to the IVH group, the IVH + TNF-α inhibition group showed reduced expression of pNF-κB (P = 0.042) and AQP1 (P = 0.030) proteins in the CPEs, while the expression of AQP-4 protein increased (P = 0.035). There were no significant differences between the two groups in the expression of NF-κB (P = 0.168), IL-1β (P = 0.235), or NKCC1 (P = 0.965) proteins. Compared to the IVH group, the IVH + NF-κB inhibition group showed reduced expression of NF-κB (P = 0.027), pNF-κB (P < 0.001), AQP1 (P = 0.009), and NKCC1 (P = 0.026) proteins in the CPEs, while the expression of AQP4 protein increased (P = 0.001). There was no significant difference in IL-1β protein expression (P = 0.237) between the two groups.

On day 7 post-modeling, compared to the aCSF group, the IVH group showed no statistically significant differences in the expression of NF-κB (P = 0.822), pNF-κB (P = 0.585), AQP1 (P = 0.377), AQP-4 (P = 0.235), IL-1β (P = 0.275), or NKCC1 (P = 0.443) proteins. Compared to the IVH group, the IVH + TNF-α inhibition group showed no statistically significant differences in the expression of NF-κB (P = 0.938), pNF-κB (P = 0.956), AQP1 (P = 0.639), AQP-4 (P = 0.233), IL-1β (P = 0.141), or NKCC1 (P = 0.635) proteins. Compared to the IVH group, the IVH + NF-κB inhibition group showed reduced expression of NKCC1 (P = 0.044) and IL-1β (P = 0.047) proteins, while there were no statistically significant differences between the two groups in the expression of NF-κB (P = 0.461), pNF-κB (P = 0.107), AQP1 (P = 0.533), or AQP-4 (P = 0.077) proteins.

#### Periventricular tissue.

**Fig 8:**
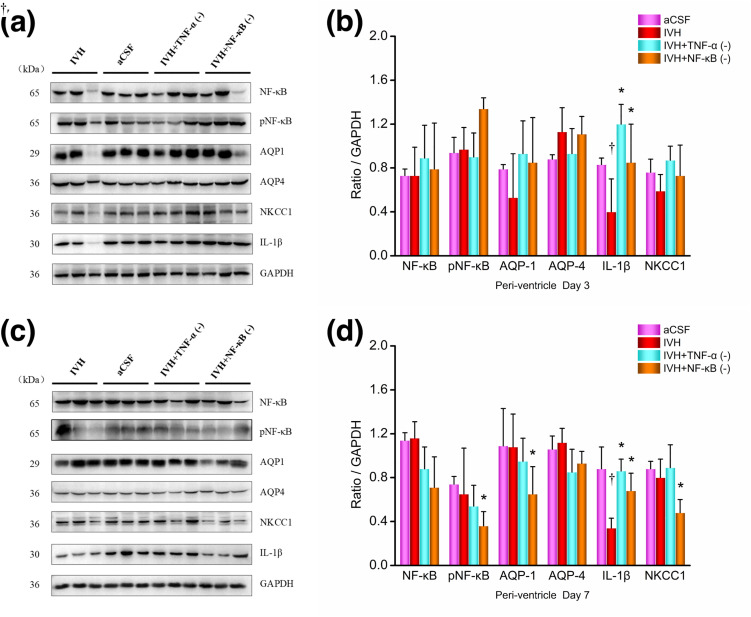
Western blot images and results of periventricular tissue proteins. (a) + (b) Day 3; (c) + (d) Day 7. n = 3; *P < 0.05 vs. the IVH group; † P < 0.05 vs. the aCSF group. CPEs, choroid plexus epitheliums; CSF: cerebrospinal fluid; aCSF: artificial cerebrospinal fluid; IVH: intraventricular hemorrhage; TNF-α: tumor necrosis factor-α; pNF-κB: phosphorylated nuclear factor κB; AQP: aquaporins; NKCC1: Na ⁺ –K ⁺ –Cl ⁻ co-transporter 1.

The results of the WB analysis of the periventricular tissue 3 days after modeling were shown in Fig 8A-B. Compared to the aCSF group, the IVH group showed decreased expression of IL-1β (P = 0.035) in the periventricular tissue, while there were no significant differences in the expression of NF-κB (P = 0.986), pNF-κB (P = 0.815), AQP1 (P = 0.345), AQP4 (P = 0.126), or NKCC1 (P = 0.293) proteins. Compared to the IVH group, the IVH + TNF-α inhibition group showed increased expression of IL-1β (P = 0.012) in the periventricular tissue, while there were no significant differences in the expression of NF-κB (P = 0.514), pNF-κB (P = 0.606), AQP1 (P = 0.169), AQP4 (P = 0.214), or NKCC1 (P = 0.097) proteins. Compared to the IVH group, the IVH + NF-κB inhibition group showed increased expression of IL-1β (P = 0.001) in the periventricular tissue, while there were no significant differences in the expression of NF-κB (P = 0.799), pNF-κB (P = 0.071), AQP1 (P = 0.254), AQP4 (P = 0.911), or NKCC1 (P = 0.361) proteins.

The results of the protein WB analysis of the periventricular tissue 7 days after modeling were shown in Fig 8C-D. Compared to the aCSF group, the IVH group showed decreased expression of IL-1β (P = 0.002) in the periventricular tissue, while there were no significant differences in the expression of NF-κB (P = 0.892), pNF-κB (P = 0.629), AQP1 (P = 0.940), AQP4 (P = 0.635), or NKCC1 (P = 0.560) proteins. Compared to the IVH group, the IVH + TNF-α inhibition group showed increased expression of IL-1β (P = 0.003) in the periventricular tissue, while there were no significant differences in the expression of NF-κB (P = 0.114), pNF-κB (P = 0.598), AQP1 (P = 0.491), AQP4 (P = 0.062), or NKCC1 (P = 0.476) proteins. Compared to the IVH group, the IVH + NF-κB inhibition group showed reduced expression of pNF-κB (P = 0.020), AQP1 (P = 0.045), and NKCC1 (P = 0.034) proteins in the periventricular tissue, while IL-1β protein expression was increased (P = 0.023), and there were no significant differences in the expression of NF-κB (P = 0.177) or AQP4 (P = 0.179) proteins.

### Immunofluorescence detection

#### CPEs on Day 3.

**Fig 9:**
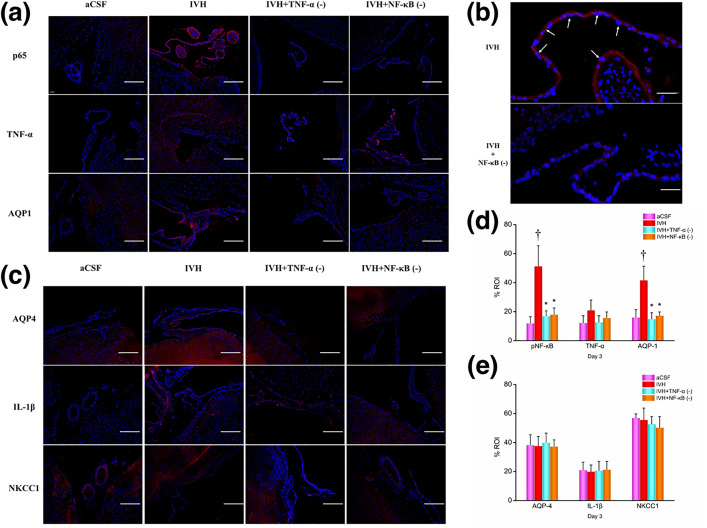
Immunofluorescence images and results of CPEs on day 3 post-modeling. (a) 100x magnification: p65 (pNF-κB), TNF-α, AQP-1; (b) p65 (pNF-κB), scale bar = 100 μm; (c) 100x magnification: AQP-4, IL-β, NKCC1; (d)+(e) results analysis. n = 3; *P < 0.05 vs. the IVH group; † P < 0.05 vs. the aCSF group. CPEs, choroid plexus epitheliums; CSF: cerebrospinal fluid; aCSF: artificial cerebrospinal fluid; IVH: intraventricular hemorrhage; TNF-α: tumor necrosis factor-α; pNF-κB: phosphorylated nuclear factor κB; AQP: aquaporins; NKCC1: Na ⁺ –K ⁺ –Cl ⁻ co-transporter 1. The arrows indicate cells expressing the target proteins. Scale bars indicate 100 um.

The protein expression levels in CPEs at 7 days post-modeling is illustrated in Fig 9. Compared to the aCSF group, the expression of p65 and AQP1 proteins in the CPE of the IVH group was significantly increased, while there was no difference in the expression of TNF-α, AQP4, NKCC1, and IL-1β proteins. Compared to the IVH group, the expression of p65, TNF-α, and AQP1 proteins in the CPE of the IVH + TNF-α inhibitor and IVH + NF-κB inhibitor groups was reduced, while there were no differences in the expression of AQP4, NKCC1, and IL-1β proteins.

#### CPEs on Day 7.

**Fig 10:**
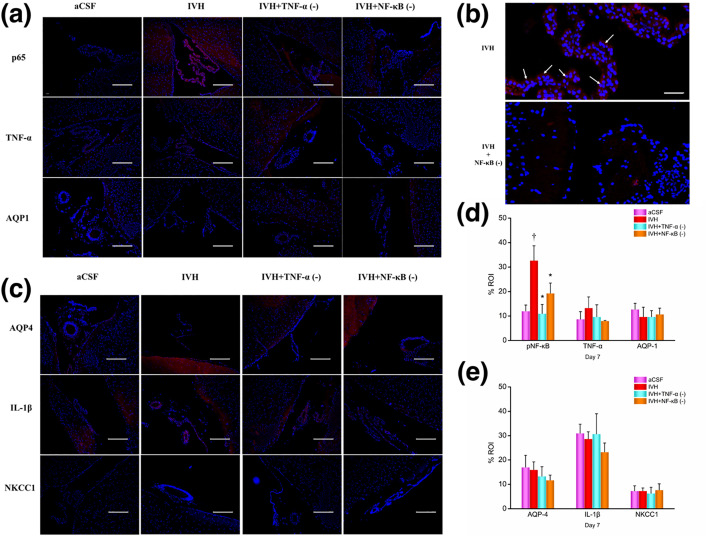
Immunofluorescence images and results of CPEs on day 7 post-modeling. (a) 100x magnification: p65 (pNF-κB), TNF-α, AQP-1; (b) p65 (pNF-κB), scale bar = 100 μm; (c) 100x magnification: AQP-4, IL-β, NKCC1; (d)+(e) results analysis. n = 3; *P < 0.05 vs. the IVH group; † P < 0.05 vs. the aCSF group. CPEs, choroid plexus epitheliums; CSF: cerebrospinal fluid; aCSF: artificial cerebrospinal fluid; IVH: intraventricular hemorrhage; TNF-α: tumor necrosis factor-α; pNF-κB: phosphorylated nuclear factor κB; AQP: aquaporins; NKCC1: Na ⁺ –K ⁺ –Cl ⁻ co-transporter 1. The arrows indicate cells expressing the target proteins. Scale bars indicate 100 um.

The protein expression levels in CPEs at 7 days post-modeling is illustrated in Fig 10. The data reveal that, compared to the aCSF group, the IVH group exhibited significantly higher expression of p65 in CPEs than both the IVH + TNF-α inhibitor and IVH + NF-κB inhibitor groups. No significant differences in the expression levels of TNF-α, AQP1, AQP4, IL-1β, and NKCC1 were observed among the different groups.

## Discussion

The clinical trial results indicate that CSF diversion surgery is not the optimal treatment for patients with PHH. Consequently, subsequent research should focus on the inflammatory response following hemorrhage, aiming to elucidate its role in hydrocephalus development and the mechanisms through which inflammation is regulated. This research aims to identify non-invasive, effective targeted drug therapies.

In this part of the study, we conducted a prospective clinical investigation to measure the concentrations of inflammatory cytokines TNF-α, IL-1α, IL-1β, IL-6, and IL-10 in the CSF of patients. Early studies have already shown elevated levels of inflammatory cytokines in the CSF and peripheral blood of PHH patients [23]. Several studies have demonstrated alterations in cytokine levels in the CSF of newborns with PHH, including TNF-α, interferon-γ (IFN-γ), IL-1β, IL-6, IL-8, IL-10, IL-12, TGF-β1, and TGF-β2 [24–26]. Turner et al. [27] recently found that cytokines such as TNF-α, IL-1α, IL-1β, IL-6, IL-8, and IFN-γ provide pro-inflammatory signals, while IL-10 and IL-12 act as anti-inflammatory molecules by inhibiting cytokine production and activating natural killer cells. TGF-β1 is often considered a specific biomarker for PHH. In the early stages of IVH, platelets rapidly aggregate and release TGF-β1, which can lead to scarring and fibrosis in the ventricular system and subarachnoid space, thereby affecting the normal circulation of CSF [28]. Experimental evidence suggests that inhibiting TGF-β1 can prevent the progression of PHH [29].

Additionally, recent CSF proteomic analyses by Yurashevich et al. [23] identified downregulation of complement C3 and coagulation factors as predictors of post-neurosurgical inflammation, providing new biomarkers for PHH risk stratification. Liu et al. [20] further confirmed that gestational age < 32 weeks and high-grade IVH are independent predictors of PHH in preterm infants, emphasizing the need for early intervention. Our study also observed elevated levels of inflammatory cytokines in the CSF of adults with chronic PHH. Compared to control groups (healthy individuals and patients who did not develop hydrocephalus following hemorrhage), the concentration of TNF-α was significantly higher in the CSF of patients with post-hemorrhagic communicating hydrocephalus, while other cytokines (IL-1α, IL-1β, IL-6, IL-10) showed no significant differences.

### Protein expression of TNF-α and NF-κB in the CPEs following IVH

TNF-α is a crucial cytokine involved in the activation of the immune system. It can be produced by activated macrophages as well as by various other cell types, including lymphocytes, mast cells, endothelial cells, myocardial cells, adipocytes, connective tissue cells, and neurons [30]. In the central nervous system, TNF-α is produced by microglia and astrocytes, promoting inflammatory cascades [31,32]. Studies using IVH animal models have demonstrated that inhibition of TNF-α post-IVH can alleviate inflammatory cell infiltration, apoptosis, neuronal degeneration, and gliosis in the periventricular region [33]. Recent research on acute-phase IVH patients revealed that TNF-α levels in the CSF rapidly increase within 1–2 days after IVH, before quickly declining and stabilizing [11]. These observations suggest that in the chronic phase of IVH, TNF-α, acting as an upstream signal of inflammation, may elevate within the ventricular system, thereby exacerbating inflammation and leading to ventricular expansion and hydrocephalus.

To validate this hypothesis, subsequent studies focused on the potential mechanisms by which TNF-α mediates and activates inflammatory responses, as well as its role in hydrocephalus formation, using a PHH rat model. Our findings indicated that on day 3 post-IVH, the CSF secretion rate significantly increased. However, by days 7 and 14, the secretion rate markedly decreased, though it remained higher than the control group. MRI analysis showed no significant difference in lateral ventricle volume between the IVH and aCSF groups at day 3. However, at day 7 post-hemorrhage, the lateral ventricle volume in the IVH group was significantly larger than in the control group, and MRI did not reveal any narrowing or obstruction of the fourth ventricle. The lateral ventricle volume did not differ significantly between days 3 and 7 in the IVH group, whereas the aCSF group exhibited a smaller lateral ventricle volume at day 7 compared to day 3. This suggests that short-term ventricular expansion in the aCSF group was not due to increased CSF secretion. Previous studies have also observed that ventricular injection of aCSF leads to ventricular expansion. Karimy et al. [3] proposed that impaired meningeal lymphatic (mLVs) drainage contributes to IVH-induced inflammation, while Lin et al. [7] demonstrated that TLR4/NF-κB blockade with TAK-242 attenuates ventricular enlargement.

Our research demonstrates that increased CSF secretion post-IVH is a primary cause of ventricular expansion. The secretion rate slows over time, but ventricular expansion persists, likely due to fibrosis in the subarachnoid space, narrowing, and obstruction of the subarachnoid space, and impairment of CSF circulation. Additionally, Yang et al. [34] revealed that glymphatic dysfunction exacerbates Aβ deposition in PHH, suggesting that ventricular expansion may result from the leakage of blood metabolites or CSF into surrounding brain tissue, leading to reduced brain tissue compliance [35].

Further analysis of inflammatory cytokine concentrations in the CSF of rats revealed a significant increase in TNF-α levels on day 3 post-IVH compared to the controls. Literature indicates that blood-derived metabolites such as methemoglobin can stimulate TNF-α production by periventricular microglia and astrocytes, thereby inducing inflammatory responses [36]. Inflammation plays a critical role in the pathogenesis and progression of various neurological disorders, including traumatic brain injury, brain tumors, and stroke. Similarly, IVH leads to inflammatory responses in the brain, which may correlate with the development of hydrocephalus post-IVH. **Li** et al. [12] showed that nitazoxanide (NTZ) suppresses neuroinflammation via dual JAK2/STAT3 and NF-κB inhibition, highlighting its potential as a repurposed drug for PHH. Early studies have identified subarachnoid and pia mater fibrosis, and ependymal proliferation, which may lead to subarachnoid space fibrosis and inflammatory changes in the arachnoid granulations, obstructing CSF circulation and causing hydrocephalus [37–39].

### Mechanisms underlying increased CSF production following NF-κB pathway activation

In our ongoing research, we have further investigated the mechanisms by which activation of the NF-κB signaling pathway leads to increased CSF production. Since approximately 99% of CSF is water, and about 80% of CSF is produced by the choroid plexus, the secretion of CSF is largely dependent on the water transport processes of the choroid plexus, which involve both water production and reabsorption [40–42]. Various proteins involved in regulating water transport in the choroid plexus have been identified in both human and animal models, including aquaporins and co-transporters [43–45]. On the apical membrane of the choroid plexus, potassium-chloride co-transporter 4 (KCC4) works synergistically with the Na + /K+−ATPase to secrete water from the choroid plexus into the ventricular system [46]. NKCC1 is also located on the apical membrane of the choroid plexus. However, Xu et al. [47] established that NKCC1 mediates CSF clearance during early postnatal development, while Wang et al. [35] linked HMGB1/JAK1/STAT3 signaling to NF-κB activation in metabolic-inflammation crosstalk.

Aquaporin 1 (AQP1) is another protein considered crucial for CPEs water transport, predominantly expressed in the apical membrane of the mammalian choroid plexus [48]. AQP1 facilitates bidirectional water transport, not only secreting water from the choroid plexus into the ventricular system (thus contributing to CSF production) but also reabsorbing water into the bloodstream. Its transport of water molecules is primarily regulated by the osmotic pressure of the CSF [49]. High expression of AQP1 has been observed in choroid plexus tumors [50]. Conversely, AQP1 knockout mice show significantly reduced CSF production [51]. These studies underscore the critical role of AQP1 in CSF secretion and maintaining water-ion balance in the choroid plexus.

In our study, we found that three days after IVH, AQP1 expression in the CPEs was significantly increased. A similar increase in AQP1 expression was observed in the aCSF group, potentially due to pressure changes in the ventricular system following aCSF injection, which could alter osmotic pressure and subsequently increase AQP1 expression, leading to water reabsorption into the bloodstream. Notably, Deng et al. [17] demonstrated that SIRT1-mediated deacetylation of NF-κB/p65 reduces neuronal apoptosis, suggesting epigenetic regulation as a therapeutic avenue. NKCC1 expression in CPEs did not show significant changes after IVH, and its expression decreased following NF-κB inhibition, indirectly suggesting that NKCC1 does not play a major role in CPEs water transport after IVH. However, modulation of the NF-κB signaling pathway appeared to influence NKCC1 expression.

Additionally, our study observed a decrease in AQP4 expression in CPEs three days after IVH. AQP4 is generally considered to be primarily expressed in ependymal cells surrounding the ventricles [52]. Previous research has shown that AQP4 protein expression is elevated in ependymal cells in chronic hydrocephalus rat models, facilitating the absorption of CSF from the ventricular system into the capillaries of the brain interstitial tissue [53]. Increased AQP4 expression in intracerebral hemorrhage (ICH) rat models has been associated with enhanced brain tissue edema [54]. Other studies have demonstrated AQP4’s role in maintaining the integrity of the ependymal cell layer. Li et al. [55] compared wild-type mice with AQP4 knockout mice and found that AQP4 knockout mice had reduced ventricular volumes, decreased CSF production, increased brain tissue water content, and disrupted ependymal structure. Feng et al. [56] reported that approximately 10% of AQP4 knockout mice developed hydrocephalus, characterized by ependymal structure disruption and obstructed CSF circulation. Our study revealed reduced AQP4 expression in CPEs after IVH, with no significant changes in AQP4 expression in the surrounding ventricular tissue. Further investigation is needed to elucidate the underlying causes and mechanisms.

The findings indicate that activation of the NF-κB signaling pathway after IVH leads to increased CSF production through the upregulation of AQP1 protein expression, mediated by inflammatory responses in CPEs. Given the critical role of the NF-κB pathway in the development and progression of inflammation, recent studies have explored targeting this pathway to mitigate inflammation and improve disease outcomes. Xiao et al. [5] identified that oxytocin inhibits neuronal pyroptosis via OXTR/p-PKA/DRP1 signaling after germinal matrix hemorrhage, while Li et al. [9] confirmed that interferon therapy suppresses TRAF3-dependent NF-κB activation. Similarly, our study further investigates the effects of NF-κB pathway inhibition on inflammatory responses following IVH. Administering TNF-α and NF-κB inhibitors every 8 hours after IVH (up to 48 hours post-hemorrhage) resulted in reduced CSF production and ventricular enlargement, and decreased NF-κB and AQP1 expression in CPEs compared to the IVH group. These results suggest that early inhibition of the NF-κB pathway after IVH can reduce CSF production and mitigate ventricular enlargement, consistent with previous studies demonstrating the positive effects of NF-κB inhibition in IVH and PHH rat models.

The observed decrease in TNF-α levels following NF-κB inhibition in our study appears to contradict the canonical signaling pathway where TNF-α acts upstream of NF-κB. However, this observation can be explained by the positive feedback loop between TNF-α and NF-κB that is well-documented in inflammatory diseases, including those affecting the central nervous system [30–32]. While TNF-α is a potent activator of NF-κB, NF-κB itself can transcriptionally upregulate TNF-α expression. This bidirectional crosstalk creates a self-amplifying inflammatory cycle that can exacerbate tissue injury. In the context of IVH model, initial hemorrhage-induced release of TNF-α likely activates NF-κB, which in turn sustains and amplifies TNF-α production. Thus, when we inhibit NF-κB with PDTC, we interrupt this feedback loop at a critical convergence point, leading to reduced TNF-α expression despite its upstream position in the initial activation cascade. Supporting this mechanism, studies in cerebral hemorrhage models have demonstrated that inhibition of NF-κB signaling significantly downregulates TNF-α expression.

**Fig 11:**
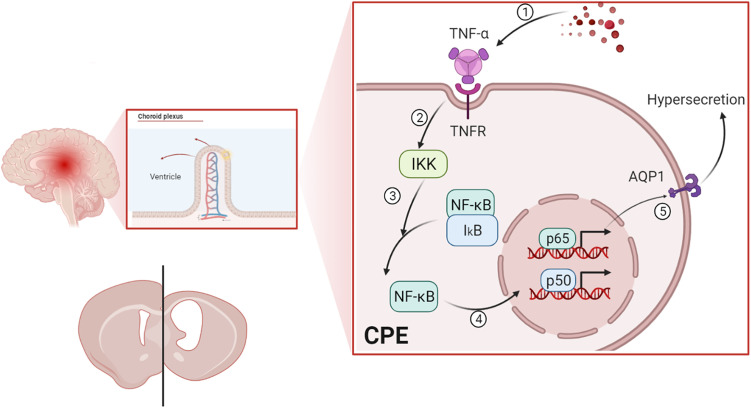
Mechanism of hydrocephalus formation involving inflammation after IVH. Following IVH, blood-related metabolites promote a significant increase in TNF-α levels in CSF. This elevation leads to the binding of TNF-α to its receptor on the CPEs membrane, triggering the activation of IKK in the cytoplasm. NF-κB (p65) dissociates, becomes activated, and translocates into the nucleus, initiating the expression of target genes. Upon activation of the NF-κB pathway, AQP1 expression is upregulated, facilitating the excessive transport of water from the choroid plexus to the ventricular system. This results in increased CSF secretion and a marked dilation of the lateral ventricles in rats. When NF-κB is inhibited after ventricular hemorrhage, a significant reduction in the size of the bilateral lateral ventricles is observed, along with a slowed CSF secretion rate and decreased expression of NF-κB and AQP1 proteins in CPEs. CPEs, choroid plexus epitheliums; CSF: cerebrospinal fluid; aCSF: artificial cerebrospinal fluid; IVH: intraventricular hemorrhage; TNF-α: tumor necrosis factor-α; pNF-κB: phosphorylated nuclear factor κB; AQP: aquaporins.

Additonally, it is important to note a complex aspect of our inflammatory data regarding IL-1β. An intriguing finding in our study was the apparent discrepancy in IL-1β levels measured by different techniques. While we observed a significant decrease in IL-1β protein in the periventricular tissue of the IVH group via Western blot (Fig 8), which was conversely increased in the inhibitor groups, no significant changes were detected in CSF by ELISA (Fig 6) or in choroid plexus epitheliums (CPEs) via immunohistochemistry (Figs 9 and 10). This inconsistency can be plausibly explained by the two-signal mechanism governing IL-1β secretion. The production and release of mature, bioactive IL-1β is a tightly regulated, two-step process: the first signal (priming), often provided by NF-κB activation, upregulates the transcription and translation of pro-IL-1β (the inactive precursor). The second signal (activation), typically mediated by inflammasome complexes, cleaves pro-IL-1β into its active form via caspase-1, leading to its secretion [57]. In our IVH model, the hemorrhage likely provided a strong priming signal, leading to the accumulation of pro-IL-1β within the cells of the periventricular region. However, the inflammasome activation signal might have been quiescent or sub-threshold, resulting in inefficient conversion and release of mature IL-1β into the CSF. Consequently, the total IL-1β protein detected by Western blot in tissue lysates (which includes both precursor and mature forms) showed a decrease post-IVH, possibly reflecting rapid turnover or degradation of untranslated precursor. The subsequent increase in the inhibitor groups could be attributed to complex feedback mechanisms or a shift in the cellular response upon pathway inhibition. The lack of significant change in CSF IL-1β (ELISA) and CPEs (IHC) further supports the notion that the inflammasome-mediated release of IL-1β was not robustly triggered in our specific model context and time frame Fig 11.

## Conclusions

Based on our findings, we propose a mechanism by which NF-κB signaling pathway activation after IVH mediates CPEs inflammatory responses and contributes to hydrocephalus formation, as illustrated in Fig 4−2. After IVH, there is significant ventricular enlargement, accelerated CSF production, and elevated TNF-α levels in the CSF. TNF-α binds to receptors on the CPEs, activating IKK in the cytoplasm, leading to the dissociation and activation of NF-κB (p65), which then translocates to the nucleus to initiate target gene expression. Activation of the NF-κB pathway upregulates AQP1 expression, resulting in excessive water transport from the choroid plexus to the ventricular system and increased CSF production. Early inhibition of NF-κB after IVH reduces ventricular enlargement, slows CSF production, and lowers NF-κB and AQP1 expression in CPEs. Thus, targeting the NF-κB pathway may offer a promising strategy for the prevention and treatment of PHH.

## Supporting information

S1 Data (XLSX)

S1 File (PDF)
