# Supplementary figures and images for: Inhibition of NF-κB pathways alleviates hydrocephalus via modulation of choroid plexus epithelium inflammation in a rat intraventricular hemorrhage model
Source: PLoS One. 2025 Nov 21;20(11):e0336346. doi: 10.1371/journal.pone.0336346 (PMC12637893; doi:10.1371/journal.pone.0336346)

Figure 7 (a)

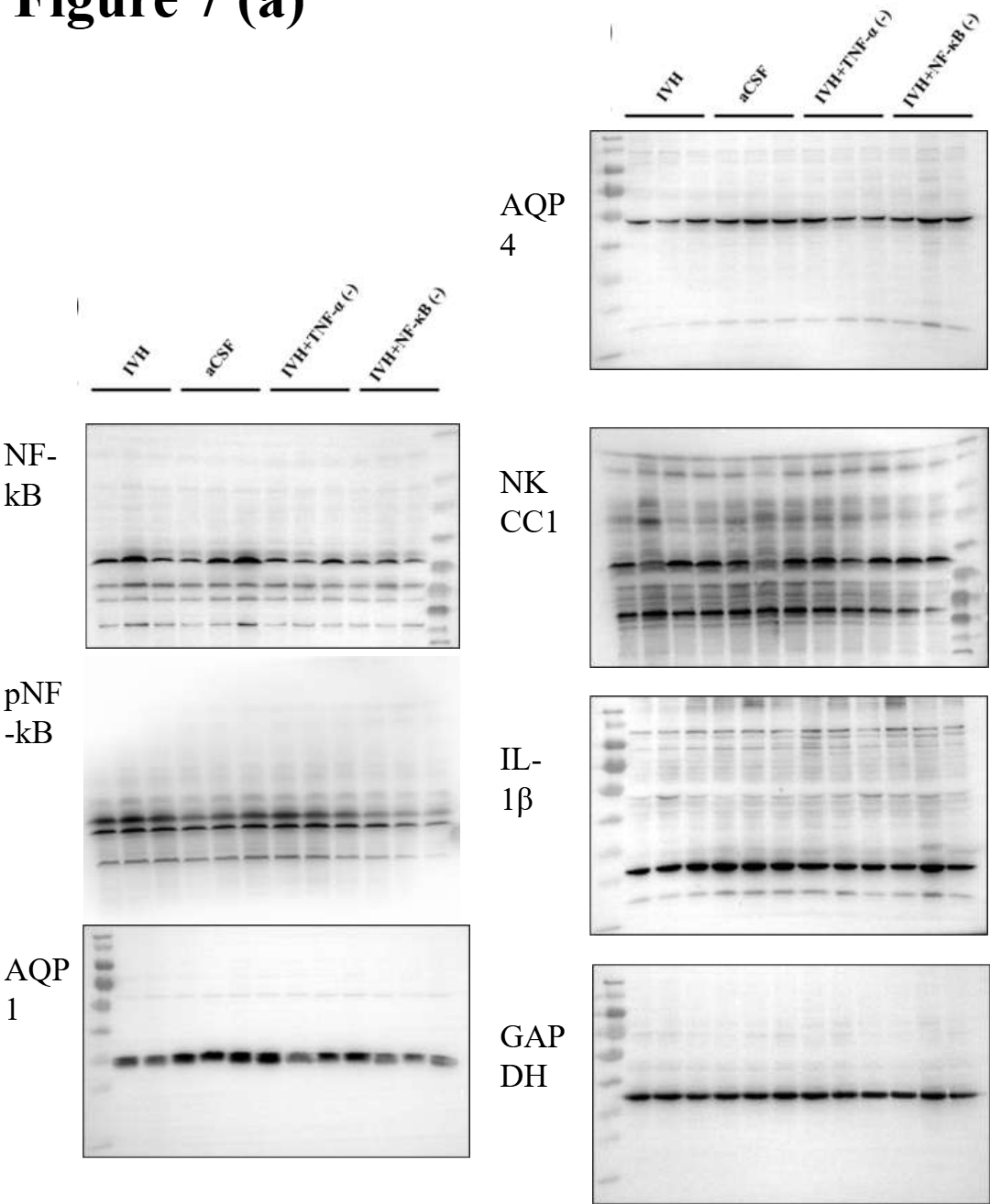

Figure 7 (c)

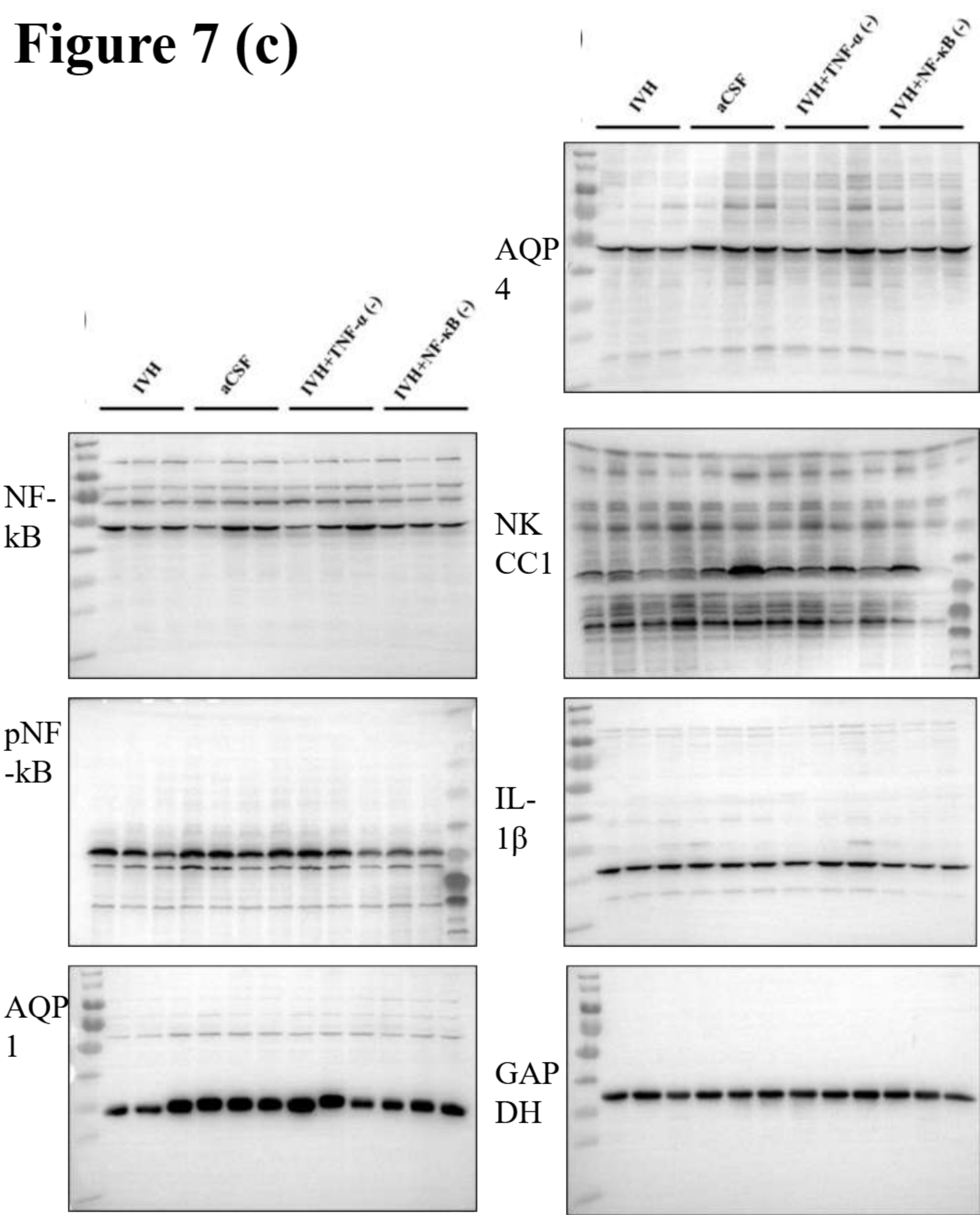

Figure 8 (a)

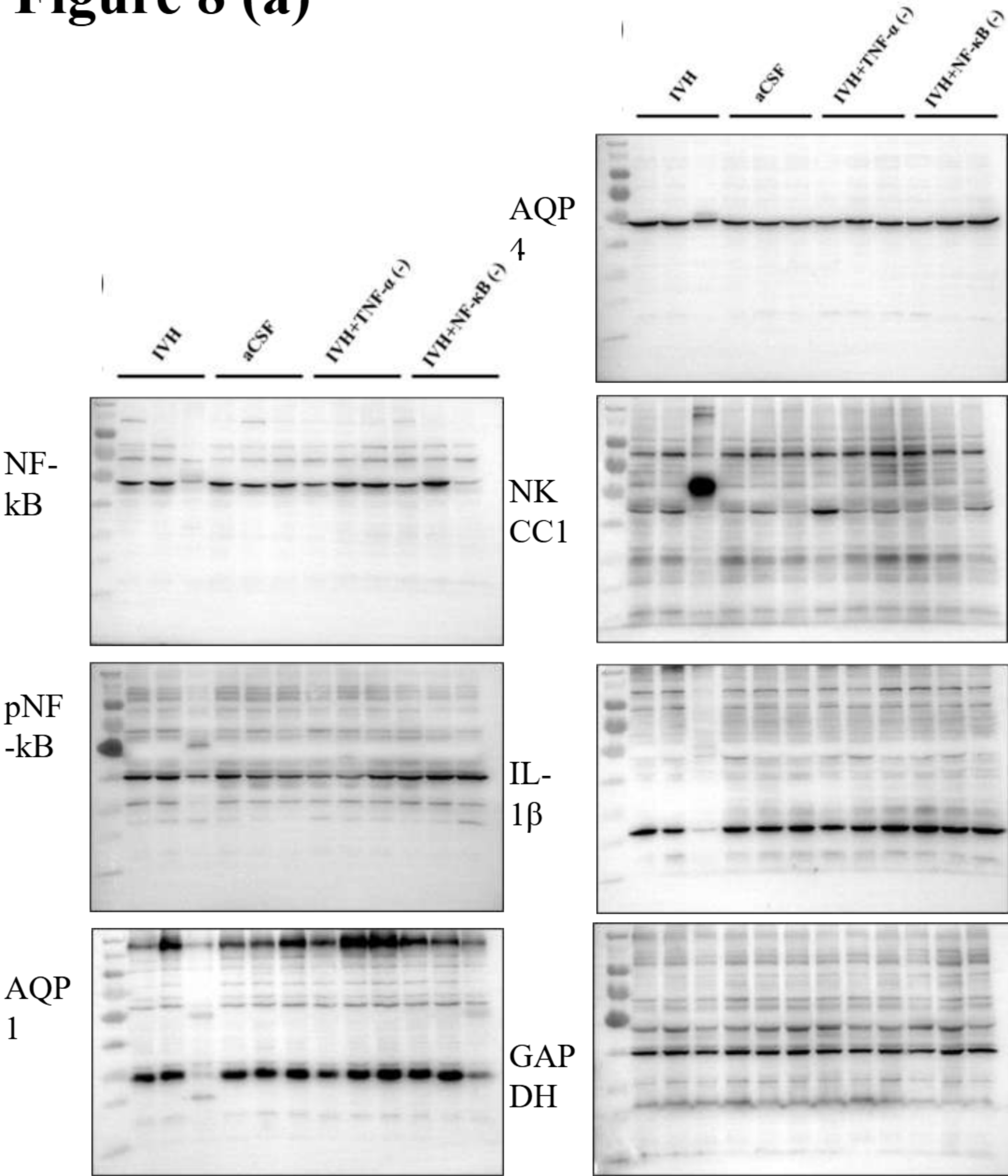

Figure 8 (c)

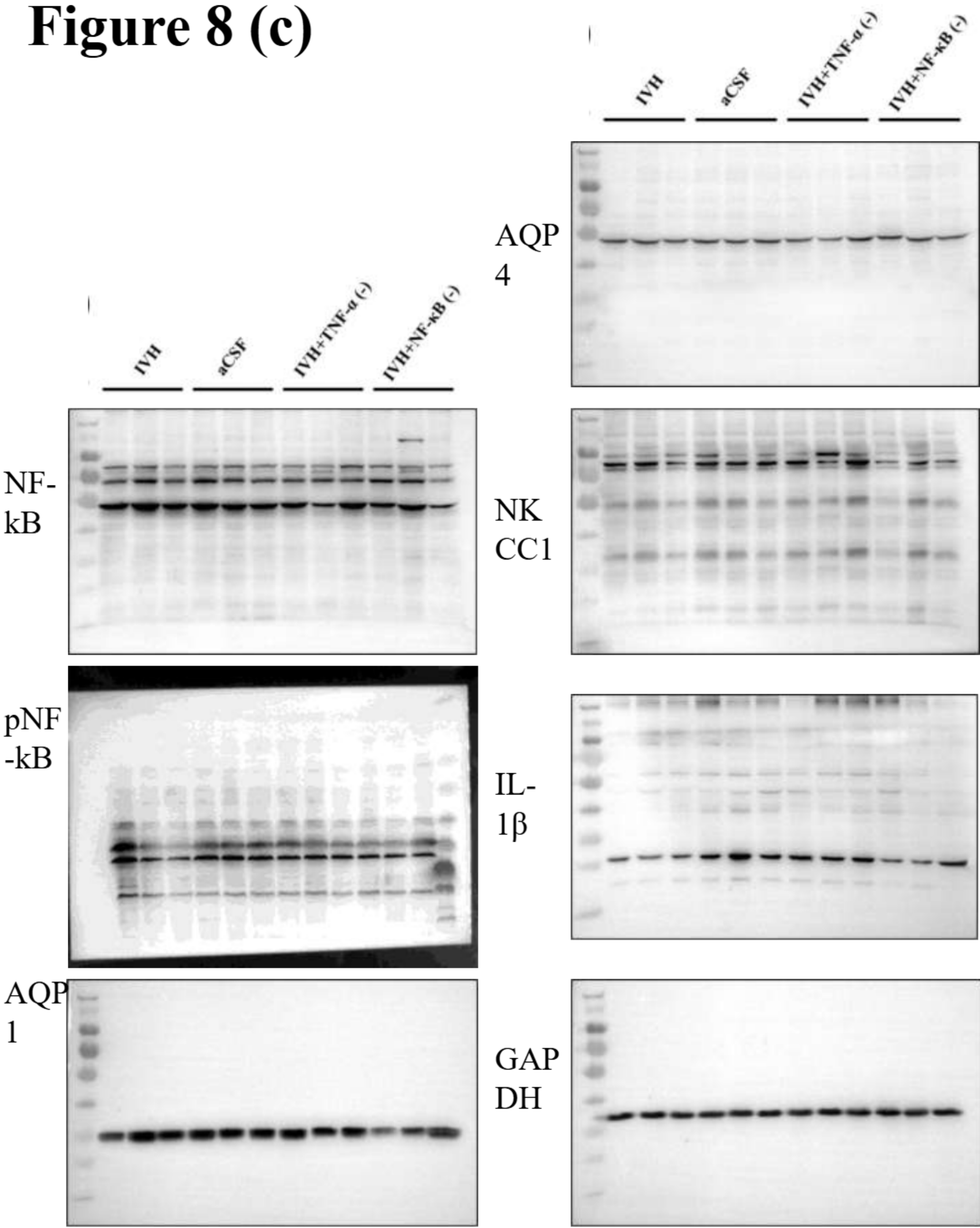

Supplement: S1 File — (PDF) [file pone.0336346.s002.pdf]
